# Two-Dimensional Aortic Size Normalcy: A Novelty Detection Approach

**DOI:** 10.3390/diagnostics11020220

**Published:** 2021-02-02

**Authors:** Paolo Frasconi, Daniele Baracchi, Betti Giusti, Ada Kura, Gaia Spaziani, Antonella Cherubini, Silvia Favilli, Andrea Di Lenarda, Guglielmina Pepe, Stefano Nistri

**Affiliations:** 1 Department of Information Engineering, University of Florence, 50139 Florence, Italy; paolo.frasconi@pm.me (P.F.); daniele.baracchi@unifi.it (D.B.); 2 Department of Experimental and Clinical Medicine, University of Florence, 50139 Florence, Italy; betti.giusti@unifi.it (B.G.); ada.kura@unifi.it (A.K.); guglielmina.pepe@unifi.it (G.P.); 3 Atherothrombotic Diseases Center, Careggi Hospital, 50139 Florence, Italy; 4 Marfan Syndrome and Related Disorders Regional (Tuscany) Referral Center, Careggi Hospital, 50139 Florence, Italy; 5 Department of Pediatric Cardiology, Meyer Hospital, 50139 Florence, Italy; gaia.spaziani@meyer.it (G.S.); silvia.favilli@meyer.it (S.F.); 6 Cardiovascular Center, Azienda Sanitaria-Universitaria Integrata of Trieste, 34125 Trieste, Italy; antonella.cherubini@asugi.sanita.fvg.it (A.C.); dilenar@units.it (A.D.L.); 7 Cardiology Service, CMSR Veneto Medica, 36077 Altavilla Vicentina, Italy

**Keywords:** echocardiography, thoracic aorta, machine learning, normalcy, Z-score, sinuses of Valsalva

## Abstract

**Background:** To develop a tool for assessing normalcy of the thoracic aorta (TA) by echocardiography, based on either a linear regression model (Z-score), or a machine learning technique, namely one-class support vector machine (OC-SVM) (Q-score). **Methods:** TA diameters were measured in 1112 prospectively enrolled healthy subjects, aging 5 to 89 years. Considering sex, age and body surface area we developed two calculators based on the traditional Z-score and the novel Q-score. The calculators were compared in 198 adults with TA > 40 mm, and in 466 patients affected by either Marfan syndrome or bicuspid aortic valve (BAV). **Results:** Q-score attained a better Area Under the Curve (0.989; 95% CI 0.984–0.993, sensitivity = 97.5%, specificity = 95.4%) than Z-score (0.955; 95% CI 0.942–0.967, sensitivity = 81.3%, specificity = 93.3%; *p* < 0.0001) in patients with TA > 40 mm. The prevalence of TA dilatation in Marfan and BAV patients was higher as Z-score > 2 than as Q-score < 4% (73.4% vs. 50.09%, *p* < 0.00001). **Conclusions:** Q-score is a novel tool for assessing TA normalcy based on a model requiring less assumptions about the distribution of the relevant variables. Notably, diameters do not need to depend linearly on anthropometric measurements. Additionally, Q-score can capture the joint distribution of these variables with all four diameters simultaneously, thus accounting for the overall aortic shape. This approach results in a lower rate of predicted TA abnormalcy in patients at risk of TA aneurysm. Further prognostic studies will be necessary for assessing the relative effectiveness of Q-score versus Z-score.

## 1. Introduction

The thoracic aorta (TA) is a geometrically complex structure that is routinely assessed by standard two-dimensional (2D) transthoracic echocardiography (TTE) [1,2]. Aortic dilatation is an important predictor of outcome, and its detection prompts the need for appropriate clinical and imaging follow-up [3,4,5]. Thus, an accurate definition of normalcy and the availability of tools for assessing normalcy are crucial for diagnosis and follow-up strategies. Although a large body of literature exists on normal limits of TA size, findings in previous studies are not immediately comparable because of different demographic and anthropometric characteristics of the study populations, different TTE modes [M-mode vs. 2D], and different strategies for measurements, including interfaces (leading-to-leading vs. inner- to-inner) and timing (end-systole vs. end-diastole) [6,7,8,9,10,11,12]. Moreover, the usability of the results of these studies for practical purposes may be limited since some of them assessed the aortic size at only one level of the TA (usually the sinuses of Valsalva). Furthermore, most studies provide ranges of normal limits or a graphical approach to assess normalcy, while only some provide algorithms to predict normalcy on a single patient basis taking into account the influence of demographics and anthropometrics on TA size resulting in a Z-score calculator. With one notable exception [12], the majority of available algorithms are only applicable to specific cases: on one hand, those assessing the aortic size at each of the four TA levels are confined to neonates, infants and young adults [6,8], or provide graphic nomograms separated for sex, body size and age groups [7]; on the other hand, most of those describing only the aortic root are based on different echocardiographic conventions for childhood and adulthood [5,8]. This may also result in some difficulties in choosing different calculators in certain ages (e.g., patients in the age range 15–18) with potential inconsistent results based on different algorithms.

Many previous approaches to establish nomograms of aortic diameters are based on linear regression modeling via ordinary least squares. Diameters are predicted from demographic and/or anthropomorphic variables such as height, weight, age, and sex, or derived measure of body size such as body surface area (BSA) and body mass index (BMI). Once fitted, these models produce normalcy calculators based on Z-scores. Despite the advantages of using Z-scores, several limitations of these approaches to normalcy have been discussed [13,14,15]. In summary, the relationship between predictors and a certain aortic diameter is not necessarily linear. Similarly, assumption of a constant variance (homoscedasticity) of the dependent variable across the entire range of the independent variable is either violated or not accounted for (further details in the Supplementary Materials file).

In this paper, we develop a general tool for TA normalcy measured at four levels of the ascending aorta by 2D-TTE in a large group of healthy individuals ranging from pediatric to elderly. For this purpose, we considered two alternative approaches: a conventional approach based on linear regression models, and a novelty detection approach based on a machine learning technique known as one-class support vector machine (OC-SVM) [16]. Novelty detection techniques are common in the context of industrial applications like, for example, in discovering frauds, in surveillance, or in monitoring and detecting structural defects of industrial assets [17]. Novelty detection has also found a few successful applications in some medical contexts like the identification of mass-like structures in mammograms [18] or detecting seizures from intracranial electroencephalogram [19]. However, to the best of our knowledge, they have never been applied before in the context of TA normalcy. We validated and compared the two approaches using two independent cohorts of subjects with expected increased prevalence of TA dilatation either due to the presence of Marfan syndrome (MFS) or related disorders, or bicuspid aortic valve (BAV) or with aortic diameter >40 mm at any TA level [1,4,20,21].

## 2. Materials and Methods

### 2.1. Study Population

This study was conducted according to the guidelines of the Declaration of Helsinki. Ethical review and approval were waived for this study, since only anonymized data, from database produced during routinely clinical activities, were used, after informed consent. Healthy individuals aged 5 years or older, with appropriate image quality for proximal aorta analysis, were prospectively and consecutively identified and enrolled during clinical activity in 3 independent echocardiographic laboratories from February 2014 to November 2017, if they had normal 12-lead ECG, left ventricular (LV) ejection fraction ≥ 55%, normal wall motion score index, and normal right ventricular size and function. Subjects were excluded if any of the aortic levels could not be visualized or if they were first-degree relatives either of patients with bicuspid aortic valve (BAV) or TA aneurysm/dissection, or Marfan syndrome (MFS) or related disorders. They were also excluded if pregnant, or participants to agonistic sport activities, or based on multiple criteria (Supplementary Materials file). As independent validation cohorts, we enrolled subjects deemed-at-risk of TA dilatation due to either clinical diagnosis of MFS and related disorders by the revised Ghent criteria [21], or BAV, and outpatients with aortic diameter >40 mm at any TA levels studied in the same laboratories.

### 2.2. Echocardiography

Comprehensive echocardiographic examinations were prospectively performed using commercially available systems equipped with a multifrequency phased-array transducer according to a predefined protocol for the performance of the echocardiographic exam, its storage, review and measurement, by 3 board-certified cardiologists with >10 years of clinical experience, during ECG-monitoring(G.S. for all the pediatric subjects (<14 years)-Florence-iE33 (Philips Medical Systems, Andover, MA, USA) A.C.-Trieste-and S.N.-Altavilla Vicentina-Vivid 7 (GE Vingmed Ultrasound, Horten, Norway)).

The aortic diameters were measured in 2D-TTE mode at 4 levels [i.e.: aortic annulus (AAn), sinuses of Valsalva (SoV), sinotubular junction (SJ), and proximal ascending aorta (PAA)]. The parasternal long-axis views were optimized to align the echocardiographic plane with each of the 4 aortic levels to obtain the largest aortic diameters; magnified views were used for greater precision. All measurements were made at end-diastole, perpendicular to the long axis of the aorta, using the leading-edge to leading-edge technique for all levels but AAn, for which inner-to-inner technique was used. Measurements were made in 3 to 5 consecutive cardiac cycles and averaged.

Height (in m) and weight (in Kg) were measured at the time of the TTE; body mass index (BMI) was computed as weight/height squared and body surface area (BSA) calculated by the Du Bois and Du Bois formula as BSA = Weight ^0.425^ × Height ^0.725^ × 0.007184. Systolic and diastolic blood pressures were measured using a properly sized cuff sphygmomanometer at the end of the examination.

To assess reproducibility, the main investigator (SN) repeated the analysis after a period of 2 weeks. A second independent observer, blinded to principal observer’s results, performed the measurements in a randomly chosen subgroup of 50 subjects from each laboratory. Interobserver variability was studied as intraclass correlation coefficients (ICCs).

### 2.3. Regression Analysis Model

Classic multivariate regression analysis was employed to predict aortic diameters from age, sex, and BSA. As in previous approaches [3,5,6,7,8,10,11,12], the data generation process was assumed to be homoscedastic and the mean squared error on training data was used to estimate the variance and to derive Z-scores. Unlike previous approaches that focused on specific age groups, and trained separate sex-specific models, a single model was trained on all the available data in healthy individuals, yielding a single normalcy calculator that is applicable to any individual for the 4 different aortic levels.

### 2.4. One-class Support Vector Machine Model

**Figure 1:**
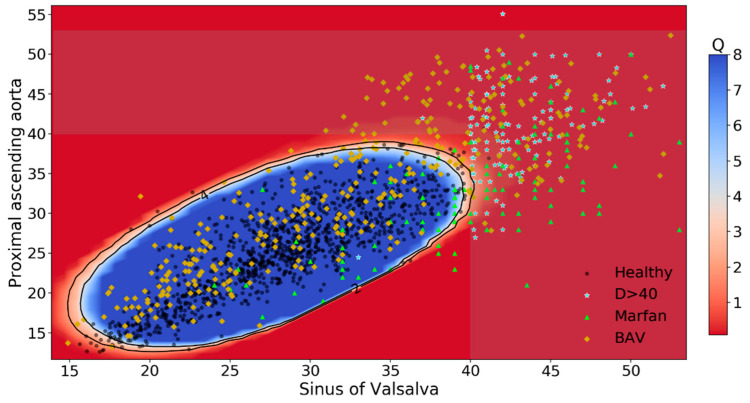
Heatmap of Q-score obtained by an OC-SVM model. As an illustrative example, this heatmap of Q-score predicting normalcy by using only proximal ascending aorta (PAA) and sinuses of Valsalva (SOV) diameters (blue dots) is reported. As a reference, the thoracic aorta dilatation region [defined as diameter (D) > 40 mm for at least one aortic segment] has been slightly darkened, and patients represented as stars. Inside the blue region, Q-scores are > 4% indicating normalcy. The black solid contour lines correspond to the level sets Q-score = 2% and 4%. Orange triangles are patients with Marfan syndrome, green diamonds patients with bicuspid aortic valve (BAV).

The one-class support vector machine (OC-SVM) [17] is a machine learning method that estimates the support (i.e., the high-density region) of an unknown joint probability distribution over a given set of variables. Given a dataset of instances drawn from this distribution and a desired percentile value ν the learning algorithm infers a function that maps a given vector of observations *x* to a real number *f_ν_*(*x*) whose sign is associated with normalcy, i.e., only a fraction ν of data points will have *f_ν_*(*x*) < 0 while a positive value for *f_ν_*(*x*) is achieved when *x* is in the support of the distribution (see mathematical details in the supplementary materials), indicating normalcy. Note that in OC-SVM only the sign of *f_ν_*(*x*) has a clear semantics, while its absolute value is not calibrated. In order to define an interpretable normalcy score, we define the Q-score of *x* as the smallest value for which *f_ν_*(*x*) < 0. This is obtained in practice by training separate OC-SVM models for all possible percentiles ν in the range [0.01, 0.3] at increments of 0.01. In our context, *x* is a vector representing age, sex, BSA, and a single or all 4 aortic diameters as our goal is to estimate normalcy of the combination of these features. Individual variables were normalized in the interval [0, 1]. When *x* contains age, sex, BSA and one individual aortic diameter, we obtain a local Q-score (i.e., relative to the particular chosen diameter). When *x* includes all four diameters we obtain a global Q-score. To the best of our knowledge, the global Q-score is the first normalcy indicator that takes into account the whole aorta morphology. Since Q-score is a continuous quantity expressing percentiles (see also Figure 1), any choice of a specific cutoff value is somewhat arbitrary. Under the assumption that the conditional distribution of a diameter, given demographic and anthropomorphic variables, is Gaussian, a (one-sided) Z-score of 2 approximately translates into the 2.3 percentile; hence, we capture approximately the same fraction of the population by assigning definite abnormalcy when the Q-score is below 2%. Taking into account the continuous nature of Q-score, we suggest 4% as a precautionary threshold (i.e., borderline abnormalcy). Unlike linear regression (that results in one coefficient for each variable and one intercept) the nature of the OC-SVM model makes it difficult to report in the paper the coefficients computed by the training algorithm. For this reason, we made the calculator freely available at http://aorta-normalcy.unifi.it. For comparison, the calculator also reports the Z-score computed by means of a linear regression model trained on our data. Further details on the model, and a discussion illustrating additional technical differences between OC-SVM and linear regression can be found in the Supplementary Materials.

In the impossibility to represent the seven-dimensional feature space of our OC-SVM model (4 aortic levels, BSA, age and sex), as an illustrative example, Figure 1 shows a heatmap of Q-score obtained by a OC-SVM model that predicts normalcy by using only PAA and SoV diameters. As a reference, the TA dilatation region defined by the current guidelines (>40 mm for at least one aortic segment) has been slightly darkened. The black solid contour lines correspond to the level sets Q-score = 2% and 4%. Points outside the 2% region have low probability, potentially indicating abnormalcy. 

### 2.5. Age Correction Option

OC-SVM detects outliers using implicitly the joint distribution of all variables. Since elders (age > 75 years) are rare in our sample of healthy subjects (*n* = 16, 1.4%), advanced age by itself becomes an important feature to determine abnormalcy. For this reason, we propose a slightly modified model where age *A* is corrected according to the following formula:

$$C=75\left(1-{\left(\frac{90-min\left\{A,90\right\}}{90}\right)}^{2}\right)$$

where *C* is the new variable used to train and test the model.

### 2.6. ROC Analysis

Receiver operating characteristic (ROC) curves were used to assess and compare the discriminatory power of the Z-score and the Q-score in distinguishing between normal and (possibly) pathological individuals. Significance in the pairwise comparisons between the areas under the curve (AUC) was assessed by a DeLong test [22] using the pROC package [23]. AUC 95% confidence interval was calculated by Bootstrap approach. For each model, the best cutoff that maximized the sensitivity-specificity sum was determined and gives an indication of the optimal model’s sensitivity and specificity. A value of *p* < 0.05 was chosen as the cut-off level for statistical significance.

## 3. Results

### 3.1. Characteristics of Study Cohort

**Figure 2:**
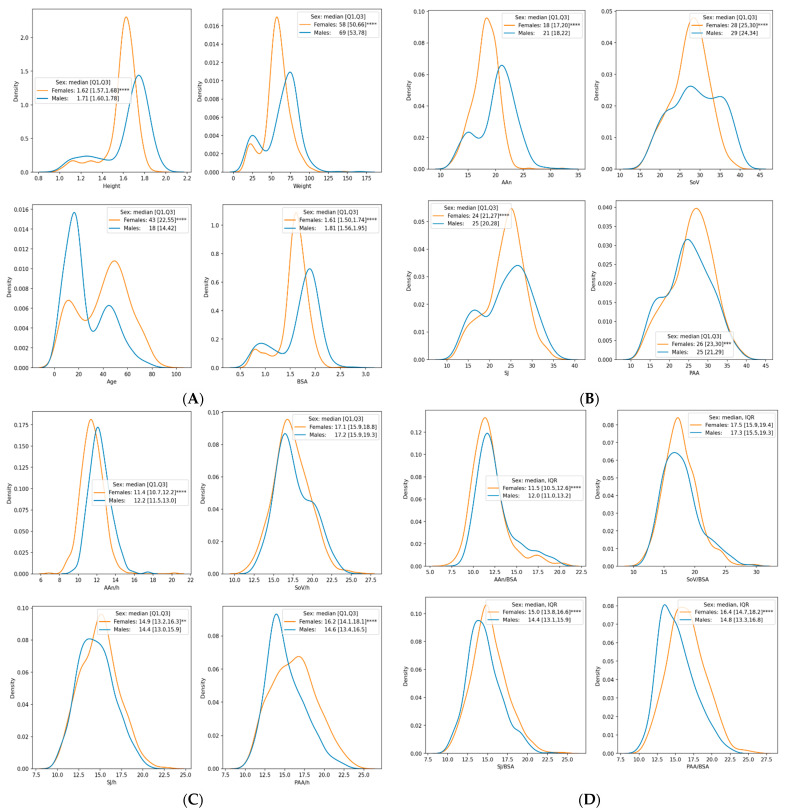
Kernel density estimates of demographics, anthropometrics and aortic sizes by sex. Distribution of height, weight, age and body surface area (BSA) are in (**A**). Distribution of aortic size at the annulus (AAn), sinuses of Valsalva (SoV), sino-tubular junction (SJ) and proximal ascending aorta (PAA), are reported as raw values (**B**) and after indexation for height (h; **C**) and BSA (**D**). (**** *p* < 0.0001; *** *p* < 0.001; ** *p* < 0.01 (Kolmogorov–Smirnov two-sided test)).

We enrolled 1112 healthy subjects satisfying our enrollment criteria, aging from 5 to 89 years (Table 1), mostly female (*n* = 586, 52.7%). Two-hundred fifty seven (23.1%) were ≤15 years, 325 (29.2%) were from 16 to 35 years, 355 (31.9%) were from 36 to 55 years, and 175 (15.7%) were ≥ 56 years. While females were older than males, height, weight, and BSA were significantly lower in females than in males (Figure 2A). Only 4 subjects had BMI > 30 kg/m^2^. We also identified (*A*) 198 patients with increased aortic size, i.e., >40 mm in at least one aortic level [1], and (*B*) 466 patients deemed-at-risk due to either MFS [21] (*n* = 115), or patients with isolated congenital BAV (*n* = 351). Normal aortic sizes, raw and indexed for BSA, are reported in Table 2 as median and IQR. Reproducibility of the aortic measurements at the four TA levels (AAn, SoV, SJ, PAA), both in children and adults, was good to excellent without significant differences among the participating echo-labs. In children, ICC for intra-observer reproducibilities at each level were 0.94, 0.98, 0.95, 0.98 respectively, while ICC for inter-observer reproducibilities were 0.9, 0.98, 0.94, 0.97. In adults, ICC for intra-observer reproducibilities at each TA level were 0.9, 0.96, 0.95, 0.9 respectively; ICC for inter-observer reproducibilities were 0.88, 0.96, 0.94, 0.92. 

Aortic diameters were larger in men compared with women at AAn, SoV and SJ; however, PAA was larger in women (Figure 2B). After indexing for height, men showed statistically significant larger aortic size at AAn, while female displayed larger diameters at SJ and PAA; non-significant trend was present at SoV (Figure 2C). Similarly, after indexing aortic diameters to BSA, dimensions of the proximal aorta were larger in women at SJ and PAA, larger in males at AAn, while no sex-related difference was evidenced at SoV (Figure 2D). 

**Figure 3:**
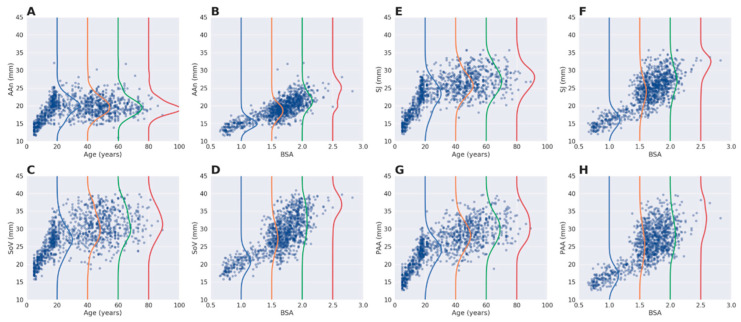
Scatterplots of individual patient aortic size related to age and body size. Individual patient data are reported to illustrate the relationship between either age or body surface area (BSA) and aortic size at the annulus (AAn; **A**,**B**), sinuses of Valsalva (SoV; **C**,**D**), sino-tubular junction (SJ; **E**,**F**) and proximal ascending aorta (PAA; **G**,**H**). Colored lines (or curves) represent the distribution of the variance of aortic size for the given values of age and BSA.

On average, aortic size was larger at SoV than at PAA with a difference (Delta-Ao, in mm) that was higher in males than in females. Noteworthy, age did not significantly affect Delta-Ao in males, while in females Delta-Ao changed along different age groups (Supplementary Materials Figure). For consistency with the practical aims of our study in clinical echocardiography and for introducing a lower number of variables in the model without losing information, we utilized age, sex, and BSA for subsequent analyses (Table 3). All the investigated parameters significantly affected aortic size at each of the four aortic levels. The effect of age was predominant at each aortic level before 20 years, and was negligible afterward only for AAn (Figure 3A,C,E,G). BSA was a significant determinant of each of the four aortic level, with a particularly large scatter of distribution for SoV, SJ and PAA (Figure 3B,D,F,H).

**Figure 4:**
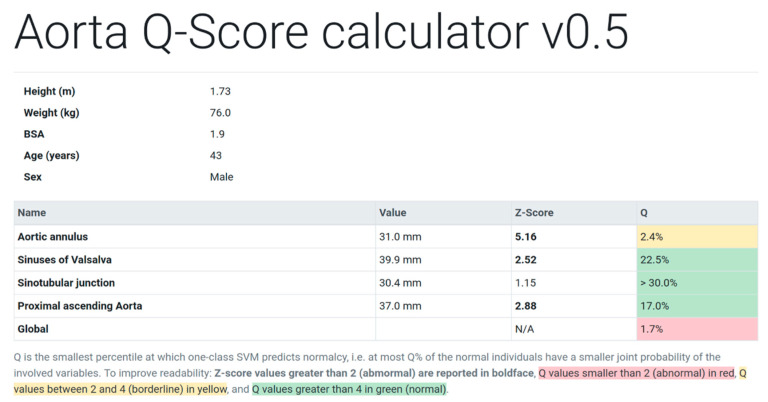
Outputs of both Z-score and Q-score in one patient. Representative output using the web calculator in a 43 year-old male with mildly steno-insufficient bicuspid aortic valve. The first two columns report the different aortic levels and their respective size in mm. The third column reports results of the Z-score for each aortic level, while the last one provides the regional and global Q-scores. Z-score values greater than 2 (abnormal) are in bold. Q-score values smaller than 2% (abnormal) are highlighted in red, while values between 2% and 4% (borderline) are colored in yellow. Q-values greater than 4% (normal) are in green.

Based on this multivariate regression analysis a Z-score calculator for each of the 4 TA levels is provided (link above). Taking into consideration the same variables, a Q-score calculator for every single TA region, and a global Q-score, considering the interplay among different aortic diameters is also provided (link). In Figure 4, the outputs of both methods are represented. 

### 3.2. ROC Analysis Comparing Z-Scores and Q-Scores

As a first step, we performed a ROC analysis discriminating 198 adult individuals considered to definitively have TA dilatation (defined as >40 mm for at least one aortic segment) from healthy subjects, using a stratified 10-fold cross validation procedure. The AUC of the global Q-score model including sex, age and BSA (0.989; 95% CI 0.984–0.993, sensitivity = 97.5%, specificity = 95.4%) was significantly higher than AUC obtained of the linear regression model, including the same variables (0.955; 95% CI 0.942–0.967, sensitivity = 81.3%, specificity = 93.3%; *p* < 0.0001). Note that, the comparisons were similar for each aortic level, both for the model trained on the overall population and after including only adults (Table 4). 

### 3.3. Behavior of Z-Score and Q-Score in Deemed-at-Risk Individuals (n = 466)

We separately calculated Z-scores and Q-scores in the cohort of individuals deemed-at-risk due either to the presence of BAV (*n* = 351) or MFS (*n* = 115). Based on the linear regression analysis, the prevalence of TA dilatation in BAV (as Z-score > 2 in at least one segment), was 243/351 (69.2%) while it was 151/351 (43.0%) as global Q-score > 4%. The prevalence of TA dilation in MFS (as Z-score > 2 in at least one segment) was 99/115 (86.1%) while it was 86/115 (74.8%) as global Q-score ≤ 4%. In Figure 4, noticeably, Z-score is abnormal at three aortic levels (AAn, SoV, PAA), while Q-score is borderline abnormal only at AAn. Importantly, however, global Q-score is definitively abnormal, capturing the joint distribution of all four diameters, thus accounting for the overall aortic shape.

One-hundred twenty patients had Z-score > 2 in at least one TA region but global Q-score > 4% (Table 5). Out of these patients, 102 had normally functioning or mildly dysfunctioning non-syndromic BAV (Table 4). The remaining 18 patients were diagnosed as MFS, of whom 11 had a genetic testing for fibrillin-1 (*FBN1*) or transforming growth factor-beta receptor-1 or -2 gene mutations. Five out of 11 were diagnosed as MFS based on abnormal Z-score; they had Q-score > 4% and a negative, comprehensive, genetic study. On the other hand, six MFS patients with a *FBN1* mutations, four of whom had familiar MFS, reached the diagnosis even in the absence of aortic compromission due to either a systemic score >7 and/or ectopia lentis. In the remaining seven patients without a genetic evaluation, three had abnormal regional Q-score at SoV. In 15 patients (3.2%) the global Q-score was <4% but Z-score was <2 (Table 4), including five patients with MFS.

## 4. Discussion

The majority of nomograms and algorithms available to predict normal size of the TA are of limited usability in a clinical echocardiographic laboratory since multiple algorithms are often needed for different age-groups and sexes. Moreover, not all of them assess normalcy at every aortic level [3,4,5,6,7,8,9,10,11,12]. Ideally, a normalcy calculator for the TA should (a) assess the aortic size at several levels (at least sinuses of Valsalva, sinotubular junction, and proximal ascending aorta); (b) predict normalcy on a single patient basis taking into account the influence of demographics and anthropometrics on TA size; (c) be uniformly applicable to a wide range of patients, thus avoiding the introduction of age, sex or body-size groups that potentially make decisions intricate for subjects situated near the range extrema, and during the follow up. 

To satisfy these needs, we developed a general and comprehensive tool for 2D-TTE normalcy of each of the four levels of TA, based on a cohort of healthy individuals over a wide age range, free from family history either of TA aneurysm/dissection, or MFS or BAV. At difference from most previous studies, women were significantly older than males. As expected [3,5,7,9,10,11,12], raw aortic diameters were larger in men compared with women at AAn, SoV and SJ, although PAA was larger in women. After indexing to BSA, women had larger SJ and PAA and smaller AAn, largely consistent with previous reports [3,7,9].

Our tool offers two alternative normalcy assessment methods, one based on the traditional linear regression model and one based on a novel strategy that employs the machine learning algorithm OC-SVM. Both models were trained on the same data derived from demographic and anthropometric characteristics of our study group of 1,112 normal individuals. Each method provides distinctively different scores, namely a Z-score and a Q-score, allowing prediction of normalcy for each aortic level. Additionally, the OC-SVM is able to provide a global Q-score, taking into account the morphology of the whole aorta (Figure 4). As a further novelty, we also verified the effectiveness of the two methods in a cohort of individuals classified as abnormal based on current guidelines [1], and in a cohort of individuals deemed-at-risk due to the presence of either MFS or BAV [4,20,21].

Noteworthy, both scores had an excellent performance in detecting abnormality in patients with TA > 40 mm. Nonetheless, estimated prevalence of TA dilatation in deemed-at-risk individuals was different between the two methods, with peculiar discordant patterns (Table 4). Intriguingly, the discrepancies between the two proposed methods occur in the strict surroundings of the normalcy borderline, where expected rate of aortic events is very low [4,20]. Indeed Milleron et al. [4] showed that, in MFS patients with TA < 50 mm, the occurrence of 1 proven or possible aortic dissection was possible after a mean of 1432 or 1718 years of follow-up, respectively. Similarly, Michelena et al. [20] demonstrated that incidence of aortic dissection in BAV was 3.1 cases per 10,000 patient-years. Thus, considering the aortic size in these discordant patients (Table 4), the hypothesis of an outcome implication of our findings in terms of acute aortic event is, by far, remote. The different performances of the two proposed algorithms on the validation cohorts lead some clinical remarks. MFS is a systemic syndrome, highly variable in symptoms and age of onset [24] whose diagnosis relies on Z-score [21]. Thus, normal Q-score depicts a MFS phenotype without relevant aortic implications [4,24]. Of clinical interest, normal Q-score does not hamper the diagnosis of MFS in our discordant patients with a positive genetic testing or in familial MFS with significant systemic features. Importantly, Q-score was >4%, in five patients with clinical diagnosis of non-familiar MFS, mostly based on abnormal Z-score, but a negative comprehensive genetic testing. 

Furthermore, in patients with non-syndromic BAV, the utilization of Q-score provides a more conservative definition of normalcy with considerable clinical implications. The absence of aortic dilatation in BAV patients, in fact, positively influences self-perception and quality of life of these patients, usually in their childhood or adolescence, and also affects medical costs reducing the frequency of follow-up evaluations. 

Though recommended, screening strategies of relatives of patients with non-syndromic TA aneurysm are affected by heterogeneous criteria used for defining aortic dilatation and by uncertainty regarding effectiveness, costs and psychological impact [25]. Q-score could find application as a unique tool for these screening programs, over a wide age-range, for each aortic level, and providing a global assessment. The long-term effectiveness of this approach needs to be assessed in prospective studies on well characterized populations, possibly including genetic testing, to define a proper genotype-phenotype correlation.

Overall, the OC-SVM approach offers several advantages in comparison with conventional approaches: (a) it does not rely on any assumption on the density of interest and it is therefore not affected by problems such as heteroscedasticity and residuals that are not normally distributed; (b) it can exploit a kernel function to measure the similarity between a new test subject and the subjects in the training population; kernel functions implicitly map a realization x to a point in an infinite dimensional feature space, thus allowing to model complex nonlinear relationships among variables; (c) as mentioned above, the global Q-score can take into account the interplay among aortic diameters (thus incorporating global morphological information of the whole aorta), by including them in x together with all other demographic and anthropomorphic variables.

We acknowledge, that the present study has multiple limitations beyond that of having been performed only in individuals of Caucasian descent. The echocardiographic examinations were not analyzed in a core-lab. To substantially reduce potential inconsistencies, an accurate echocardiographic protocol was agreed and prospectively performed by three expert echocardiographers. Moreover, reproducibility of the entire set of aortic measurements was comparable between echo-labs. We believe that this procedure, together with strict inclusion criteria, may have limited the potential negative influence of multiple readers on our findings and their interpretations. Stress testing was not systematically included in the clinical protocol to rule-out exercise-induced abnormalities. However, since patients were included during clinical activity, it may have been performed when appropriate. We believe that also considering the clinical profile delineated by our inclusion criteria, the possibility of a latent disease was really negligible in our cohort. Due to the distinctively very strict enrollment criteria, our healthy group >55 years is relatively small, consistently with multiple previous studies some of which, moreover, included patients with systemic arterial hypertension [3,7,12]. 

## 5. Conclusions

We provide a new tool to assess TA normalcy and detect aortic dilatation based on a novel strategy that employs the machine learning algorithm OC-SVM, supplying the new Q-score. This model requires less assumptions about the distribution of the relevant variables compared with the Z-score. In particular diameters do not need to depend linearly on anthropometric measurements and the joint distribution of these variables together with all four diameters can be captured simultaneously, thus accounting for the overall aorta shape. Compared with the traditional linear regression model, the OC-SVM was slightly but significantly more effective in detecting abnormality in individuals with threshold-defined aortic dilatation, and provided a distinctively smaller prevalence of abnormal aortic size in patients at risk of TA dilatation due to the presence of MFS or BAV. Further prognostic studies will be necessary to assess the relative effectiveness of Q-score vs. Z-score.

## Figures and Tables

**Table 1:** Demographic and echocardiographic characteristics of the 1112 healthy subjects.

Characteristics	Median (Q1–Q3)
Age (years)	34 (16–50)
Sex F n (%)	586 (52.7)
Height (cm)	165 (157–173)
Weight (Kg)	62 (51–74)
Body mass index (Kg/m^2^)	21.99 (19.53–25)
Body surface area (m^2^)	1.68 (1.51–1.87)
Systolic blood pressure (mm Hg)	120 (110–130)
Diastolic blood pressure (mm Hg)	70 (65–80)
Heart rate (beats per minute)	72 (65–82)
Left atrial volume index (mL/m^2^)	21.0 (17.0–28.0)
Left ventricular end-diastolic volume index (mL/m^2^)	55.0 (47.0–64.0)
Left ventricular ejection fraction (%)	65 (62–68)
Left ventricular mass index (g/m^2^)	70.9 (61.0–83.0)
E/A	1.46 (1.14–1.95)
E/e’	5.9 (5.2–7.0)

E/A = mitral early diastolic velocity/atrial diastolic velocity ratio; E/e’ = mitral, early diastolic velocity/average early diastolic e’ velocity ratio.

**Table 2:** Aortic size at aortic annulus, sinuses of Valsalva, sinotubular junction and proximal ascending aorta level (raw diameter and diameter indexed for BSA) of the 1112 healthy subjects.

Aortic Levels	Raw Diameter Median (IQR)	Diameter Indexed for BSA Median (IQR)
Aortic annulus (mm)	19.1 (17.0–21.0)	11.71 (10.81–12.85)
Sinuses of Valsalva (mm)	28.0 (24.4–32.0)	17.40 (15.70–19.30)
Sinotubular junction (mm)	23.9 (21.0–27.2)	14.74 (13.42–16.24)
Proximal ascending aorta (mm)	25.9 (21.9–29.3)	15.60 (13.90–17.50)

BSA = body surface area; IQR = interquartile range.

**Table 3:** Association of age, BSA, and sex with the aortic diameter at the 4 investigated levels. Age and BSA were normalized before linear regression (Age max = 89, BSA max = 2.821).

	Aortic Annulus	Sinuses of Valsalva	Sinotubular Junction	Proximal Ascending Aorta
Intercept	0.3108 *	0.3191 *	0.2266 *	0.2234 *
Age (yrs)	0.0439 *	0.2655 *	0.2792 *	0.3563 *
BSA (m^2^)	0.5156 *	0.5132 *	0.6018 *	0.4953 *
Female Sex	−0.0525 *	−0.0600 *	−0.0297 *	−0.0150 §

*: *p* < 0.001; §: *p* < 0.05.

**Table 4:** AUC for models trained on the overall population, and only on subjects aged >15 years.

	AUC Z-Score [95%CI]	AUC Q-Score [95%CI]	*p*-Value
Overall Population			
Global	0.955 [0.942–0.967]	0.989 [0.984–0.993]	<0.0001
Sinuses of Valsalva	0.774 [0.731–0.816]	0.963 [0.950–0.977]	<0.0001
Sinotubular Junction	0.799 [0.760–0.838]	0.949 [0.933–0.964]	<0.0001
Proximal Ascending Aorta	0.908 [0.877–0.939]	0.979 [0.972–0.987]	<0.0001
Only Adults			
Global	0.947 [0.932–0.962]	0.988 [0.983–0.993]	<0.0001
Sinuses of Valsalva	0.754 [0.711–0.797]	0.957 [0.942–0.971]	<0.0001
Sinotubular Junction	0.810 [0.773–0.847]	0.960 [0.945–0.975]	<0.0001
Proximal Ascending Aorta	0.906 [0.875–0.936]	0.973 [0.962–0.983]	<0.0001

**Table 5:** Demographic and clinical characteristics of 135/466 discordant deemed-at-risk subjects based on BAV or MFS.

	Patients with Z-Score > 2 Q-Score > 4% (*n* = 120)	Patients with Z-Score < 2 but Q-Score ≤ 4% (*n* = 15)
Age, years	16.2 (12.4–36.5)	26.0 (9.2–53.0)
Age ≤ 15 years, n (%)	58 (48.3)	5 (33.3)
Sex Female, n (%)	26 (21.7)	3 (20.0)
Body surface area, m^2^	1.62 (1.27–1.82)	1.98 (1.10–2.18)
MFS, n (%)	18 (15.0)	6 (40.0)
BAV, n (%)	102 (85.0)	9 (60,0)
**Aortic size (mm)**		
Aortic annulus,	21.0 (19.0–23.7)	22.0 (16.5–24.7)
Sinuses of Valsalva	30.5 (25.0–35.0)	35.0 (24.0–38.5)
Sinotubular junction	25.0 (21.0–29.4)	28.3 (19.5–32.5)
Proximal ascending aorta	30.0 (25.5–35.0)	32.0 (23.5–34.5)
**Aortic size/body surface area (mm/m^2^)**		
Aortic annulus,	13.6 (12.4–15.6)	11.8 (11.2–16.0)
Sinuses of Valsalva	20.2 (17.8–22.4)	18.3 (16.6–22.6)
Sinotubular junction	16.5 (14.3–18.5)	16.0 (13.2–18.4)
Proximal ascending aorta	19.0 (17.6–21.7)	18.3 (15.3–21.7)

Data are median and IQR, if not otherwise specified.

## Data Availability

The datasets used and/or analyzed during the current study are available from the corresponding author on reasonable request.

## Supplementary Materials

The following are available online at https://www.mdpi.com/2075-4418/11/2/220/s1.

## References

[1] Erbel R., Aboyans V., Boileau C., Bossone E., Di Bartolomeo R., Eggebrecht H., Evangelista A., Falk V., Frank H., Gaemperli O. (2014). ESC Guidelines on the diagnosis and treatment of aortic diseases: Document covering acute and chronic aortic diseases of the thoracic and abdominal aorta of the adult. The task force for the diagnosis and treatment of aortic diseases of the European society of cardiology (ESC). Eur. Heart J..

[2] Goldstein S.A., Evangelista A., Abbara S., Arai A., Asch F.M., Badano L.P., Bolen M.A., Connolly H.M., Cuéllar-Calàbria H., Czerny M. (2015). Multimodality imaging of diseases of the thoracic aorta in adults: From the American society of echocardiography and the european association of cardiovascular imaging: Endorsed by the society of cardiovascular computed tomography and society for cardiovascular magnetic resonance. J. Am. Soc. Echocardiogr..

[3] Mirea O., Maffessanti F., Gripari P., Tamborini G., Muratori M., Fusini L., Claudia C., Fiorentini C., Pleasea E.I., Pepi M. (2013). Effects of aging and body size on proximal and ascending aorta and aortic arch: Inner edge–to–Inner edge reference values in a large adult population by two-dimensional transthoracic echocardiograph. J. Am. Soc. Echocardiogr..

[4] Milleron O., Arnoult F., Delorme G., Detaint D., Pellenc Q., Raffoul R., Tchitchinadze M., Langeois M., Guien C., Beroud C. (2020). Pathogenic FBN1 genetic variation and aortic dissection in patients with marfan syndrome. J. Am. Coll. Cardiol..

[5] Devereux R.B., de Simone G., Arnett D.K., Best L.G., Boerwinkle E., Howard B.V., Kitzman D., Lee E.T., Mosley T.H., Weder A. (2012). Normal limits in relation to age, body size and gender of two-dimensional echocardiographic aortic root dimensions in persons >/=15 years of age. Am. J. Cardiol..

[6] Gautier M., Detaint D., Fermanian C., Aegerter P., Delorme G., Arnoult F., Milleron O., Raoux F., Stheneur C., Boileau C. (2010). Nomograms for aortic root diameters in children using two-dimensional echocardiography. Am. J. Cardiol..

[7] Saura D., Dulgheru R., Caballero L., Bernard A., Kou S., Gonjilashvili N., Athanassopoulos G.D., Barone D., Baroni M., Cardim N. (2017). Two-dimensional transthoracic echocardiographic normal reference ranges for proximal aorta dimensions: Results from the EACVI NORRE study. Eur. Heart J. Cardiovasc. Imaging.

[8] Warren A.E., Boyd M.L., O’Connell C., Dodds L. (2006). Dilatation of the ascending aorta in paediatric patients with bicuspid aortic valve: Frequency, rate of progression and risk factors. Heart.

[9] Muraru D., Maffessanti F., Kocabay G., Peluso D., Dal Bianco L., Piasentini E., Jose S.P., Iliceto S., Badano L.P. (2014). Ascending aorta diameters measured by echocardiography using both leading edge-to-leading edge and inner edge-to-inner edge conventions in healthy volunteers. Eur. Heart J. Cardiovasc. Imaging.

[10] Biaggi P., Matthews F., Braun J., Rousson V., Kaufmann P.A., Jenni R. (2009). Gender, age, and body surface area are the major determinants of ascending aorta dimensions in subjects with apparently normal echocardiograms. J. Am. Soc. Echocardiogr..

[11] Bossone E., Yuriditsky E., Desale S., Ferrara F., Vriz O., Asch F.M. (2016). Normal values and differences in ascending aortic diameter in a healthy population of adults as measured by the pediatric versus adult American society of echocardiography guidelines. J. Am. Soc. Echocardiogr..

[12] Campens L., Demulier L., De Groote K., Campens L., Demulier L., De Groote K., Vandekerckhove K., De Wolf D., Roman M.J., Devereux R.B. (2014). Reference values for echocardiographic assessment of the diameter of the aortic root and ascending aorta spanning all age categories. Am. J. Cardiol..

[13] Colan S.D., Steven D. (2013). The why and how of Z scores. J. Am. Soc. Echocardiogr..

[14] Mawad W., Drolet C., Dahdah N., Dallaire F. (2013). A review and critique of the statistical methods used to generate reference values in pediatric echocardiography. J. Am. Soc. Echocardiogr..

[15] Dallaire F., Bigras J., Prsa M., Dahdah N. (2015). Bias related to body mass index in pediatric echocardiographic *Z* scores. Pediatr. Cardiol..

[16] Schölkopf B., Platt J.C., Shawe-Taylor J., Smola A.J., Williamson R.C. (2001). Estimating the support of a high-dimensional distribution. Neural Comput..

[17] Pimentel M.A., Clifton D.A., Clifton L., Tarassenko L. (2014). A review of novelty detection. Signal Proces..

[18] Tarassenko L., Hayton P., Cerneaz N., Brady M. Novelty detection for the identification of masses in mammograms. Proceedings of the 4th International Conference on Artificial Neural Networks, IET.

[19] Gardner A.B., Krieger A.M., Vachtsevanos G., Litt B. (2016). One-class novelty detection for seizure analysis from intracranial EEG. J. Mach. Learning Res..

[20] Michelena H.I., Della Corte A., Prakash S.K., Milewicz D.M., Evangelista A., Enriquez-Sarano M. (2015). Bicuspid aortic valve aortopathy in adults: Incidence, etiology, and clinical significance. Int. J. Cardiol..

[21] Loeys B.L., Dietz H.C., Braverman A.C., Callewaert B.L., De Backer J., Devereux R.B., Hilhorst-Hofstee Y., Jondeau G., Faivre L., Milewicz D.M. (2010). The revised Ghent nosology for the Marfan syndrome. J. Med. Genet..

[22] DeLong E.R., DeLong D.M., Clarke-Pearson D.L. (1988). Comparing the areas under two or more correlated receiver operating characteristic curves: A nonparametric approach. Biometrics.

[23] Robin X., Turck N., Hainard A., Tiberti N., Lisacek F., Sanchez J.-C., Müller M. (2011). pROC: An open-source package for R and S+ to analyze and compare ROC curves. BMC Bioinform..

[24] Grange T., Aubart M., Langeois M., Benarroch L., Arnaud P., Milleron O., Eliahou L., Gross M., Hanna N., Boileau C. (2020). Quantifying the genetic basis of marfan syndrome clinical variability. Genes.

[25] Mariscalco G., Debiec R., Elefteriades J.A., Samani N.J., Murphy G.J. (2018). Systematic review of studies that have evaluated screening tests in relatives of patients affected by nonsyndromic thoracic aortic disease. J. Am. Heart Assoc..

