# Supplementary material for: Two-Dimensional Aortic Size Normalcy: A Novelty Detection Approach
Source: Diagnostics (Basel). 2021 Feb 2;11(2):220. doi: 10.3390/diagnostics11020220 (PMC7912952; doi:10.3390/diagnostics11020220)
Supplement: Supplementary file 1 [file diagnostics-11-00220-s001.pdf]

Supplemental File

Details on Q-score

Our normalcy indicator, Q-score, is derived from the one-class support vector machine (OC-SVM) [17], an unsupervised learning algorithm that estimates the support of an unknown but fixed joint distribution *p* that is assumed have generated the data. By support we mean here the set of points *x* such that *p*(*x*) is greater than a given threshold. In particular, the OC-SVM algorithm receives as input a dataset of *m*-dimensional vectors $\{x_1,\ldots,x_n\}$ (in our case m=7: age, sex, BSA, and four aorta diameters) and outputs a real valued decision function *f* such that *f*(*x*)>0 if *x* belongs to the support of *p*. The method can be therefore used to divide data points in two groups. The first group contains cases that belong to the support of *p* and that are considered to be *normal*. The second group contains novel, rare, or otherwise abnormal cases. The decision function *f*(*x*) is defined as follows:

(1) $$f(x) = \sum_{i=1}^{n} \alpha_i k(x_i, x) - \rho$$

where the index *i* ranges over the subjects in the training set, *k* is a kernel function that measures the similarity between two vectors of variables, and α_i and ρ are coefficients determined by the learning algorithm. In this work we used the radial basis function (RBF) kernel, defined as

(2) $$k(x_i, x) = e^{-\gamma \|x - x_i\|^2}$$

that allows us to obtain non-linear decision functions. The RBF kernel width was set to a default value $\gamma = 1/(S\ m)$ where *S* is the standard deviation of *x* in the training set and *m* its dimensionality. The coefficients computed by OC-SVM are the solution of the quadratic problem

$$\min_{\alpha_1,\ldots,\alpha_n} \frac{1}{2} \sum_{i,j=1}^{n} \alpha_i \alpha_j k\left(\mathbf{x}_i, \mathbf{x}_j\right) \quad \text{subject to } 0 \leq \alpha_i \leq \frac{1}{\nu n}, \quad \sum_{i=1}^{n} \alpha_i = 1$$

where an important role is played by the percentile parameter ν. It is shown in [17] that ν is an upper bound to the fraction of abnormal points and asymptotically equals this fraction. For example, by setting ν=0.02 will ensure that at least 98% of the data points belong to the support of the distribution and are considered to be normal cases. By training OC-SVM on a grid of values for ν, we obtain the Q-score of a data point x, defined as the smallest percentile ν such that $f_\nu(x)<0$. The practical interpretation is that when the Q-score of a given vector of measurements x is q, then at most q% of normal individuals have measurements whose joint probability is smaller than $p(x)$.

A simplified visual example

In the impossibility to visually represent the seven-dimensional variable space of our OC-SVM model (4 aortic levels, BSA, age, and sex), as an illustrative example, Figure 1 shows a heatmap of the *Q*-score obtained by a OC-SVM model that predicts normalcy by using only two measurements: PAA and SoV diameters. Measurements for healthy, dilated, Marfan and BAV subjects are plotted in the figure. As a reference, the TA dilatation region defined by the current guidelines (>40 mm for at least one aortic segment) has been slightly darkened. The black solid contour lines correspond to the level sets *Q*-score=2% and 4%. Points outside the 2% region have low probability, potentially indicating abnormalcy. Note that level sets of Q-score delimit closed regions thanks to the use of the RBF kernel (see Eq. 2 above). By contrast, linear regression models are unable to delimit closed regions. Indeed, if we had used linear regression on the data of Figure 1, the normalcy region would have been delimited by two parallel lines, resulting in many clearly abnormal cases (e.g. patients with PAA and SoV approximately equal but both larger than 40mm) being predicted as normal.

Analytical differences between linear regression models and OC-SVM.

Regression models assume that the noise is additive and normally distributed with zero mean and constant variance (homoscedasticity). These models produce normalcy calculators based on *Z*-scores. More precisely, *Z* is calculated as the difference between the measured and the predicted diameters, divided by the standard deviation estimated from the mean squared error on the training data. The calculators are eventually used to infer the abnormality of diameters (e.g. using the criterion $Z > 2$). Several limitations of these approaches to normalcy have been discussed [14]. First, the relationship between predictors and a certain aortic diameter is not necessarily linear. To correct this, some methods assume that linearity holds in the log-log space [6] where it is proposed to predict $\log d = \beta_1 \log \text{BSA} + \beta_0$. Others have suggested the use of polynomial models [15]. A second problem is that the homoscedasticity assumption is often violated and not accounted for [14]. While methods that operate in the log-log space somewhat do take heteroscedasticity into account [6,14] (when mapping back to the original spaces, variance increases with the independent variable), the way variance varies with predictors remains constrained by the model assumptions. A third issue is that data might not satisfy the model assumptions of normally distributed additive noise [14].

Paradoxically, success and effectiveness of these methods are linked to their inaccuracy in predicting aortic diameters. Indeed, if a diameter of a new subject was predicted with a small error (by a very accurate model), then the resulting *Z*-score would be small. While this is desirable for healthy subjects, it may be problematic for patients, potentially yielding a low sensitivity when using the calculator as a diagnostic tool. Indeed, few existing normalcy studies have extensively tested the diagnostic power of the proposed calculators in terms of sensitivity and specificity, measured on an independent test set of healthy and pathological subjects.

As an advantage over conventional approaches, OC-SVM is able to delimit regions of high density with very mild assumptions about the underlying probability distribution (membership in the exponential family is sufficient), thus potentially overcoming some of the above mentioned limitations of regression models. There is finally another important advantage of OC-SVM: it allows normalcy to be assessed for the whole aorta morphology, taking all level measurements into account simultaneously, while conventional linear regression typically assesses normalcy for individual levels separately.

Exclusion criteria:

(a) arterial systemic hypertension or being on active anti-hypertensive treatment, (b) overt coronary artery disease (defined as previous acute coronary syndrome, revascularization procedures, positive stress test suggesting inducible ischemia, or coronary angiography demonstrating coronary artery stenosis >50%); (c) primary cardiomyopathy and/ or genetic cardiovascular disease; (d) congenital heart disease; (e) mitral valve prolapse, mitral or aortic valvular insufficiency of higher degree than trivial, valvular stenosis of any degree, or any previous cardiac or vascular surgery or interventional procedure (including ablation of accessory pathways); (f) previous chemotherapy and/or chest radiotherapy; (g) documented episodes of atrial fibrillation or flutter, either complex or frequent (i.e. >10 ectopic beats/hour at Holter monitoring) supra-ventricular or ventricular arrhythmias; (h) any kind of cardiovascular therapy; (i) previous cardioembolic stroke, including transient ischemic attacks; (l) diabetes mellitus or any kind of endocrinologic disorder).

Supplemental Figure: Difference between aortic size at sinuses of Valsalva and proximal ascending aorta (Delta-Ao) are reported for males (blue) and females (pink) in mm (median values as triangles, bars for IQR). Age did not significantly affect Delta-Ao in males, while in females Delta-Ao changed along different age groups.
